# Postpartum contraceptive practices among urban and peri-urban women in North India: a mixed-methods cohort study protocol

**DOI:** 10.1186/s12884-021-04294-3

**Published:** 2021-12-10

**Authors:** Nivedita Roy, Priyanka Adhikary, Rita Kabra, James Kiarie, Gitau Mburu, Neeta Dhabhai, Ranadip Chowdhury, Sarmila Mazumder

**Affiliations:** 1 Centre for Health Research and Development, Society for Applied Studies, 45 Kalu Sarai, New Delhi, 110016 India; 2 World Health Organization, Geneva, Switzerland

**Keywords:** Postpartum family planning, Mixed-method, Traditional contraception methods, Modern contraception methods, Cohort, Maternal health, Child health

## Abstract

**Background:**

Postpartum family planning (PPFP) helps women space childbirths, increase exclusive breastfeeding and prevent unintended pregnancies, leading to reduction in maternal, infant and child morbidities and mortality. Unmet need of family planning is highest among women in the postpartum period due to lack of knowledge, cultural and religious barriers, access barriers and low antenatal care service utilization. However, in spite of low prevalence of postpartum family planning practices, birth-to-birth interval is reportedly high in Delhi, India. This study explores the postpartum contraception practices and the relationship between use of postpartum contraception and subsequent child linear growth.

**Methods:**

This is a mixed method cohort study on PPFP and is nested within an ongoing “Women and Infants Integrated Interventions for Growth Study” (WINGS). Married women aged 18–30 years who have delivered a live baby are recruited for quantitative interviews at 6 weeks, 6, 12, and 24 months postpartum. In-depth interviews are conducted with a randomly selected sub-sample of women at each of the four time points, 35 husbands and 20 local service providers to understand their perspectives on PPFP practices.

**Discussion:**

The findings from the study will provide useful insights into couples’ contraception preferences and choice of contraception, modern and traditional, initiation time and the effect of birth spacing and contraception use on subsequent linear growth of the child. This knowledge will be of significant public health relevance and will help in designing appropriate interventions for appropriate postpartum contraception use and delivery strategies. The study aims to work address the Sexual and Reproductive Health and Rights goal of promoting reproductive health, voluntary and safe sexual and reproductive choices for women.

**Trial registration:**

Trial registration number: CTRI/2020/03/023954.

**Supplementary Information:**

The online version contains supplementary material available at 10.1186/s12884-021-04294-3.

## Background

One of the global public health priorities is to address the high unmet need for contraception among postpartum women in low- and middle-income countries [1, 2]. Prevention of unwanted and closely spaced pregnancies substantially reduces maternal, infant, and child mortality [3–6]. Postpartum family planning (PPFP) is defined as the initiation of contraceptive methods within the first 12 months following delivery [7, 8]. It is a high priority area and a crucial period, given the need to promote and support breast feeding, prevent unintended pregnancies, and space births, all of which contribute to reducing maternal and neonatal mortality and adverse birth outcomes [9]. Evidence shows that inter-pregnancy intervals shorter than 18 months increases the risk of adverse perinatal outcomes [10]. The World Health Organization (WHO) Technical Committee on Birth Spacing recommends an interval of at least 24 months before a couple attempts pregnancy in order to reduce the risk of adverse maternal, perinatal and infant outcomes [11]. The continuum of care throughout a woman’s pregnancy, childbirth and postpartum period provides multiple opportunities to reach her. During antenatal care (ANC), there is an opportunity to counsel and advise women on the importance of family planning (FP) and the range of contraceptive options available [12, 13]. Similarly, the postpartum period is an ideal time to initiate FP because of availability of sufficient time for counselling during hospital stay and improved ability of providers to make a holistic assessment of women’s reproductive health needs [11]. The FP 2020 Global Initiative identified PPFP within 1 year of delivery as a critical component of global FP strategy [14], considering the missed opportunities for improving reproductive and child health outcomes [15].

India is the second most populous country in the world, with half of its citizens being of reproductive age. Despite the presence of a long-standing national FP program, India has a high unmet contraceptive need and a high rate of maternal morbidity and mortality [16]. In India, 65% of the women have unmet need for FP in the postpartum period with only 26% of women using any contraception in the postpartum period [11]. Unmet need of FP is highest among women in the postpartum period [17]. Although India has experienced a decline in total fertility rate from 3.2 in 2000 to 2.3 in 2016 [16], there are wide disparities across states, rural-urban population and socioeconomic status, and there is an ongoing need for FP. Studies in India show that several obstacles prevent women from using postpartum contraception including: a lack of awareness [18–22], lack of marital communication about it [23], cultural attitudes [24], poor access to facilities [21], and poor counselling and other skills among health providers [16, 24, 25]. Even when women are aware of PPFP, studies have found a gap between their awareness and their actual utilisation due to barriers related to cultural, religious, fertility desires, duration of marriage and ANC service utilisation factors [22, 25].

However, it is to be noted that despite the generally low prevalence of PPFP in India [26, 27], evidence from the Fourth National Family Health Survey conducted in 2015–16, shows that the median (IQR) interval between two live births in Delhi is 38 (IQR 26, 60) months with 25% of women reporting an interval of up to 5 years. The survey also found that while 52% of couples were not using any contraception, the use of emergency contraceptive pills was also limited (0.32%) [28]. Therefore, factors other than use of contraception in postpartum period could be contributing to the long birth to birth interval.

Counselling for PPFP helps women to choose the contraceptive they want to use, to initiate the contraceptive use, and to continue using it, depending on the reproductive intentions of the couple [29–32]. Therefore, ensuring that all postpartum women have access to high quality FP services, in both urban and rural areas, is an important strategy for improving maternal and childhood health outcomes.

In this context, this ancillary study on PPFP, nested within an ongoing “Women and Infants Integrated Interventions for Growth Study (WINGS)” [33] provides an opportunity to explore, the contraception practices during the postpartum period among women in the WINGS.

The study aims to describe the modern and traditional contraception practices during the postpartum period till the child is 24 months old and to describe the association between use of modern and traditional contraceptives in the postpartum period and linear growth of the child at 24 months of age (attained length -for-age-z -score and stunting; LAZ score < −2SD).

## Methods

### Study design

This is a prospective, mixed method, cohort study using both quantitative and qualitative methods.

### Study setting

The study is being conducted in the urban and peri-urban low-to mid-socioeconomic neighbourhoods of South Delhi, India, where the rates of low birth weight (~ 25%), stunting (~ 40%) in children under 2 years of age and maternal undernutrition rate, i.e., body mass index (BMI) < 18.5 kg/m^2^ (22%), are similar to the national average [28].

### Study participants

In WINGS, married women, aged 18–30 years, living with their husband, having no child or one child, and wanting to have a child, are enrolled and randomized to receive the pre- and peri- conception intervention package or routine care, until they are identified to be pregnant or have completed 18 months of follow-up. Once pregnant, they are randomized for a second time either to receive the post-conception (pregnancy and postpartum) interventions or routine care [33].

Women, who deliver a live baby in WINGS, regardless of whether the birth is a singleton, twin or other higher order birth, are eligible for participation in this ancillary study. In-depth interviews are conducted with a smaller sub-sample of 35 husbands and 20 local health care providers, to understand their perceptions regarding postpartum contraception.

### Study procedures

At the time when women are first visited by the WINGS team, after giving birth, consent for sharing the contact details of women with the separate sub-study or ancillary study team, distinct from the WINGS team, is sought, mentioning that this is an independent study and not a part of WINGS. If women agree with sharing of their contact details, the separate sub-study team contacts those women at 6 weeks post-delivery, with a window period of + 10 to 14 days, when the women are comfortably settled at home, and introduce the study to them. If the woman is not comfortable or is admitted in the hospital or baby is admitted in the sick newborn care unit, the woman is not approached against her wish, under such prevailing circumstances. Women are visited consecutively at 6 weeks following delivery and new consent is sought for participation in the sub-study.

For the interviews with the husband, the woman is explained about her husband’s participation, what it would involve and whether she agrees. If the woman is comfortable and verbally consents to her husband being interviewed, the study team member explains the purpose of the study to the husband and following written informed consent, conducts the interview. If her husband is not available, his contact number is taken, and he is contacted later. Based on his convenience, the face-to-face interview is conducted, at his preferred place.

The health care providers in the study area mostly belong to the informal sector and are private practitioners in their own clinics. They do not have supervisors or higher authorities, to whom they are answerable or from whom they need to get permission. There are few government dispensaries that have nurses/Auxiliary Nurse Midwives (ANM) who provide FP services. The Medical Officer in-charge of the dispensary supervises the nurses and ANMs. Permission from the Medical Officer is obtained. A senior study team member meets the private practitioners and the government providers at their convenient time and preferred places, explains the purpose of the study, procedures and approximate time required for the interview. After obtaining written informed consent, interview is conducted.

Interviews with the husbands and male health care providers are conducted by male study team members, while the female health care providers are interviewed by female study team members. The recruitment of the participants for quantitative and qualitative components is currently ongoing.

### Sample size

#### Quantitative interviews

Prevalence of postpartum contraception use at 6 weeks and 6 months is estimated to be 10 and 21% respectively, and at 12 and 24 months is estimated to be 26% based on previous studies [18]. For evaluation of the primary outcome, the sample size estimate is based on the uptake of PPFP prevalence of 26% which results in an optimal sample. Therefore, given the 26% prevalence rate, and further assuming 20% loss to follow-up rate by 24 months, a sample size of 360 will be needed, with 5% margin of error and a two-sided 95% confidence interval. Women who delivered in WINGS and consented are recruited for the study until the target minimum sample size needed for uptake of post-partum contraception is achieved.

For evaluation of the secondary outcomes on linear child growth, the outcomes (length -for-age-z score and stunting) are restricted to those who completed the entire follow up period of 24 months. Assuming 80% evaluability (parent WINGS), approximately 288 mother-baby pairs are expected to be evaluable at 24 months and could be included in the analysis of linear child growth. Applying the FP prevalence rate of 26%, comparison of linear child growth outcomes would be between approximately 75 users and 213 non-users of postpartum contraception.

#### Qualitative interviews

Qualitative in-depth interviews are conducted with a random sample of 20 women, or until saturation, at each of the multiple time points (6 weeks, 6 months, 12 months and 24 months). This sampling is intended to provide a diverse representative sample of women’s perspectives [23]. In addition to the women, 35 husbands and 20 service providers, or until saturation, whichever is lower, will be interviewed for additional perspectives and triangulation purposes.

### Data collection

**Fig. 1:**
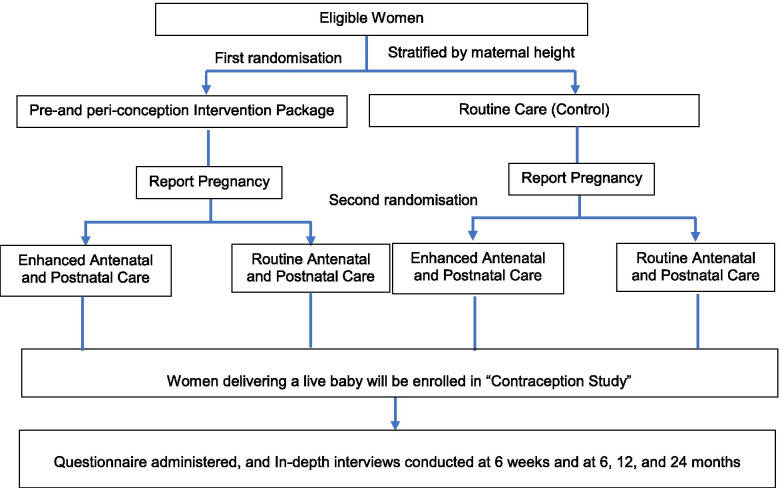
Design and Recruitment

The sub-study team comprises of well-trained personnel, with the required experience and expertise. All data for this ancillary study is collected using both quantitative and qualitative methods following the visit schedule of the parent WINGS. To minimize the burden associated with the accumulation of research processes for the participants involved in the WINGS and the ancillary study, we ensure that administration of questionnaire for both studies is not done on the same day. The WINGS procedures such as outcome measurements at 6, 12 and 24 months and some intervention activities such as counselling of mothers, are done at these points. We keep a sufficient window period of 10 to 14 days and visit the woman, only when she is comfortable. Figure 1 shows the study design and recruitment.

The quantitative questionnaire explores the sociodemographic profile of the woman and her family, contraceptive practices and repeat pregnancy in the 24 months postpartum period. Woman’s knowledge, beliefs, practices, access, barriers, service experiences, and needs regarding contraception are ascertained. Women are selected consecutively for the quantitative survey. Additionally, length of the child will be measured by the sub-study team at 24 months of child age.

Qualitative in-depth interviews are conducted to obtain insights and perspective of the women regarding their contraceptive practices. Their perceptions about lactation as a contraceptive measure, contraceptive practices, traditional or modern methods used, if any, in the postpartum period by the couple, time of initiation of FP methods following delivery with reasons, preferences of the types of contraceptives with reasons, are explored. Findings from qualitative in-depth interviews will contextualize quantitative data and inform possible interventions that could improve utilization of contraceptives.

The quantitative questionnaires and qualitative discussion guides developed for the study have been translated in the local language (Hindi) by the study investigator to ensure that the meaning of the questions is not altered. Interviews are conducted in Hindi. In the qualitative component, the information obtained from the husbands is not linked to that of the women to preserve confidentiality or to prevent reprisals. The information provides insights into the domestic environment in which women practice use of contraceptives. The interview guides are provided as Additional files 1 and 2.

Interviews with health care providers are useful to understand their perspectives, beliefs, experiences, prevalent recommendations and advice, practices, and service availability around PPFP methods. This information helps us understand the service environment in which women practice use of contraceptives.

### Data management and analysis

Quantitative and qualitative data will be analyzed and aggregated to describe contraception patterns in the postpartum period, using appropriate descriptive and summary measures.

For the evaluation of FP method uptake at 6 weeks, 6, 12 and 24 months and for FP continuation outcomes, regression analysis will be done applying the survival statistical techniques. For the linear child growth outcomes, regression analysis will be conducted to determine the risk of adverse linear growth outcomes at 24 months in children among modern method users relative to non-users, adjusting for possible confounders such as age, education, occupation, intention to have another child, duration of use, group allocation in WINGS, or other factors as identified. The predictors could include information, knowledge, provision and availability of services, access to services, religious beliefs, or influence by others.

The analysis will derive the proportions of women using PPFP and the methods used up till 24 months postpartum and association between PPFP use and linear growth outcomes in child (that is length-for-age -z-score and stunting at age 2 years).

For the qualitative data analysis, the audio-recorded in-depth interviews will be transcribed both in colloquial and English language. Provisional codes will be generated and refined, and emerging themes identified using inductive thematic analysis approach. A matrix of framework analysis will be prepared to display the themes, sub themes and respondent characteristics (case and theme-based analysis).

A code list will be generated using the information areas covered in the discussion guides i.e., prior, and open codes will be both used to analyze the content of the transcripts. All transcripts will be coded in NVivo. This coding list will then be used to code individual transcripts in NVivo using the three major pillars of any coding paradigm i.e., using code prefixes to indicate ordering, using letters to indicate sub-concepts within a ‘family of codes’ and using symbols to differentiate experiences from fact and other factors. Codes will be refined iteratively to generate final emerging themes.

### Ethical considerations

All measures are taken to ensure that woman is not under duress or any obligation and her privacy and confidentially are secured. Clear information is given so that women do not feel obligated to give consent. Woman’s autonomy is of prime importance and is ensured by the team. The interview is conducted at a place where the woman is most comfortable.

The person enrolling is a senior person of the ancillary study team, well trained, experienced, responsible, well versed with ethical issues and trained in Good Clinical Practice. The team member explains to the woman that it is completely up to the woman to participate in the study and that non-participation will not affect access to any services that she is currently receiving from any providers or from the primary study, WINGS. Only if the woman gives consent, the team member obtains written informed consent.

Participants are given a small token of appreciation, alongside counseling for FP and condoms. This is considered reasonable to compensate participants for their time.

The information collected from the study participants is kept confidential. The participants are not identified by his/her name but only by a unique identification number. All information collected is stored in a locked area with access only to the study team and study documents are stored for a period of 5 years after completion of the study. The knowledge obtained from the study will be shared through reports for the ethics review committee, government agencies and publications but none will have the name of the study participants. All data will have been aggregated before reporting. Furthermore, data will be stored in-country by SAS and only reviewed by the immediate research team.

## Discussion

Postpartum contraception knowledge and practice remains low in the postpartum period [34–37]. Studies have shown a time lag of 5 months from resumption of sex and initiation of postpartum contraception use [38]. Often Postpartum women resort to traditional FP methods, which may not suffice as the needs during this period are specific. It has been found that the perception around risk of pregnancy during lactation amenorrhea influences use of contraceptives [36, 39, 40]. There are other factors that affect postpartum contraceptive practices such as poor knowledge [41], safety of contraceptive methods and concerns around side effect [36, 40], inadequate coverage of postpartum services [13] and apprehensions of acceptance by husbands [42]. Other factors significantly associated with uptake of postpartum contraceptive device after birth included completion of secondary education, having 3–4 and ≥ 5 children, attending three ANC, ever hearing about postpartum IUCD, and having received counseling from health-care providers about the postpartum intrauterine contraceptive device [43].

There is an urgent need for effective strategies to meet women’s postpartum contraceptive needs and to understand optimal approaches. Recommendations suggested to improve utilization of PPFP services prioritize strengthening health facility delivery, promoting girls’ education and encouraging women’s participation in deciding for contraceptive use [44].

Counselling of couples, focusing on male involvement have also been suggested, although all men do not willingly accompany women to antenatal clinic [45–47]. Studies in LMICs show that a substantial proportion of women did not use modern contraceptive methods in the first year after birth and maternal services were found to be the sole predictors in postpartum contraceptive use. Findings suggest the importance of linking PPFP along the continuum of care.

It is anticipated that the study results will have some limitations in terms of generalizability, as the results can only be generalized to population with similar characteristics. The other limitation is ascertainment of contraceptive use. This will be self- reported by the women and the social response or reporting bias cannot be ruled out, unless it is an implant. Despite these limitations this study addresses an important aspect of the sexual and reproductive rights of women related to PPFP, and the findings from this study will be useful for informing potential interventions that can assist women to access and improve utilization of contraceptives in the postpartum period in similar settings.

## Conclusion

The findings emerging from the study will provide useful insights into couples’ preference and choice of contraception, initiation time and the effect of birth spacing on subsequent linear growth of the child. This knowledge will be of significant public health relevance and will help in designing appropriate interventions and delivery strategies. The study therefore aims to work towards the goal of SRHR, promoting reproductive health, voluntary and safe sexual and reproductive choices for women.

## Supplementary Information

**Additional file 1.**

**Additional file 2.**

## Data Availability

The datasets that will be used and/or analysed during the study will be available from the corresponding author on reasonable request.

## Abbreviations

ANC: Antenatal care
BMI: Body mass index
ERC: Ethics Research Committee
FP: Family planning
PPFP: Postpartum family planning
SRHR: Sexual and Reproductive Health Rights
WHO: World Health Organization
WINGS: Women and Infants Integrated Interventions for Growth Study
