# Supplementary material for: Postpartum contraceptive practices among urban and peri-urban women in North India: a mixed-methods cohort study protocol
Source: BMC Pregnancy Childbirth. 2021 Dec 10;21:820. doi: 10.1186/s12884-021-04294-3 (PMC8662907; doi:10.1186/s12884-021-04294-3)
Supplement: Supplementary file 1 — Additional file 1. [file 12884_2021_4294_MOESM1_ESM.pdf]

An ancillary study to the WINGS on post partum contraceptive practices among urban and peri urban women in North India

Quantitative Form: Postpartum Family Planning

[Code: 1=Yes, 2=No, 8=Does not know, 9=Not Applicable]

1.	WINGS ID	1.	☐☐☐☐☐☐
2.	Respondent's study ID number (eg PPFP001 to PPFP360)	2.	☐☐☐☐☐☐☐
3.	Questionnaire administered by	3.	____________________ ☐☐
4.	Consent obtained?	4.	☐
5.	Date	5.	☐☐☐☐☐☐☐☐
6.	Visit schedule (3=6 weeks, 4= 6 months, 5= 12 months, 6= 24 months)	6.	☐
7.	Marital status of the respondent (11= Married, 12=single, 13= Divorced, 14= Widowed)	7.	☐☐
8.	Age of respondent (in years)	8.	☐☐
9.	Date when you last delivered?	9.	☐☐☐☐☐☐☐☐
10.	What is the sex of your baby (3=male, 4=female)	10.	☐
11.	Was your baby born on time (3=full term; 4=preterm)	11.	☐
12.	At which gestation was baby born (months)	12.	☐☐
13.	When do you intend to have another child? Write in months	13.	☐☐
14.	How many children do you intend to have in life?	14.	☐☐
15.	Have you ever discussed with your husband on how many children he is willing to have?	15.	☐
16.	If YES, how many children does your husband prefer to have?	16.	☐☐
17.	If No, why Not?<br>____________________<br>____________________	17.	☐<br>☐

An ancillary study to the WINGS on post partum contraceptive practices among urban and peri urban women in North India

	_________________________<br>_________________________<br>_________________________		☐<br>☐<br>☐
**Knowledge of postpartum family planning (PPFP) services**			
18.	Are you aware of family planning methods?	18.	☐
19.	If YES, which are these methods?<br>Lactation Amenorrhoea Method<br>Contraceptive pills/tablets<br>Progestin-Only Pill<br>Combined Oral Contraceptive Pill<br>Cu T<br>Intrauterine Contraceptive Device<br>Injectable contraception (DMPA)<br>Implant<br>Male condom<br>Female condom<br>Natural family planning method<br>Standard Days Method<br>Sterilization (Male)<br>Sterilization (Female)	19.	 ☐<br>☐<br>☐<br>☐<br>☐<br>☐<br>☐<br>☐<br>☐<br>☐<br>☐<br>☐<br>☐<br>☐
20.	Other, please specify___________________	20.	☐
21.	Are you aware of family planning methods for postpartum mothers within the first year of delivery?	21.	☐
22.	Are you aware of family planning methods that are available at the clinic for postpartum mothers within the first year of delivery?	22.	☐

An ancillary study to the WINGS on post partum contraceptive practices among urban and peri urban women in North India

23.	If YES, which are these methods?<br>Lactation Amenorrhoea Method<br>Contraceptive pills/tablets<br>Progestin-Only Pill<br>Combined Oral Contraceptive Pill<br>Cu T<br>Intrauterine Contraceptive Device<br>Injectable contraception (DMPA)<br>Implant<br>Male condom<br>Female condom<br>Natural family planning method<br>Standard Days Method<br>Sterilization (Male)<br>Sterilization (Female)	23.	☐<br>☐<br>☐<br>☐<br>☐<br>☐<br>☐<br>☐<br>☐<br>☐<br>☐<br>☐<br>☐<br>☐
24.	Other, please specify__________________	24.	☐
25.	Are you currently using any method of contraception to delay or avoid getting pregnant?	25.	☐
26.	If NO, why Not?<br>_________________________<br>_________________________<br>_________________________<br>_________________________<br>_________________________	26.	☐<br>☐<br>☐<br>☐<br>☐
27.	If YES, which method(s) are you currently using?<br>Lactation Amenorrhoea Method<br>Progestin-Only Pill	27.	☐<br>☐

	Contraceptive pills/tablets<br>Combined Oral Contraceptive Pill<br>Intrauterine Contraceptive Device<br>Injectable contraception (DMPA)<br>Implants<br>Male condoms<br>Female condoms<br>Natural/traditional family planning methods<br>(coitus interruptus, rhythm methods, abstinence, cervical mucus)<br>Standard Days Method<br>Sterilization (Male, female)		☐<br>☐<br>☐<br>☐<br>☐<br>☐<br>☐<br>☐<br><br>☐<br>☐
28.	Other, please specify__________________	28.	☐
29.	**At 6 weeks** visit ask, When did you start using it, that is, how many days after birth of your child<br>**At 6 months, 12 months and 24 months**, ask, Since I spoke to you the last time, have you been using contraceptives	29.	☐<br>☐
30.	Have you been using continuously or off and on	30.	☐
31.	If not using continuously, for how many days didn't you use it	31.	☐
32.	Where did you receive education about family planning services<br>Labour and delivery room<br>Postnatal ward<br>Expanded programme on immunization visit<br>Home visit<br>Postnatal checkup visit<br>Well baby visits	32.	 ☐<br>☐<br>☐<br>☐<br>☐<br>☐

☐

An ancillary study to the WINGS on post partum contraceptive practices among urban and peri urban women in North India

	Friends<br>Family members/relatives<br>Health workers<br>Television<br>Newspaper		☐<br>☐<br>☐<br>☐
33.	Other, please specify__________________	33.	☐
34.	Did you visit the facility or health center for postpartum care	34.	☐
35.	If yes how many weeks after birth did you visit postpartum clinic	35.	☐☐
36.	What information about family planning was covered during your postpartum counseling?<br>No information was given<br>Return to fertility<br>Fertility intentions<br>Healthy timing and spacing of pregnancies<br>Exclusive breastfeeding<br>Lactational Amenorrhoea Method (LAM)<br>Transition from LAM to other modern methods of family planning	36.	☐<br>☐<br>☐<br>☐<br>☐<br>☐<br>☐
37.	Other, please specify__________________	37.	☐

An ancillary study to the WINGS on post partum contraceptive practices among urban and peri urban women in North India

38.	Was the information clear?<br>They did not explain well<br>They explained, but I did not understand anything<br>I felt shy to ask<br>They seemed to be so busy, did not entertain me<br>They were very rude, I did not feel like asking them<br>__________________________<br>_____________________________<br>_____________________________	38.	☐<br>☐<br>☐<br>☐<br>☐<br>☐<br>☐<br>☐
**Influence of normative beliefs of the respondent on PPFP services**			
39.	To which religious faith group or faith do you belong? (11=Christian, 12=Muslim, 13=Hindu, 14=Sikh, 15=Jain, 16=Budhhist, 17=Jewish, 18=Parsi, 19=No religion, 20=other_____)	39.	☐☐
40.	Does your denomination allow you to use modern family planning methods to limit your family?	40.	☐
41.	Does your traditional culture allow you to use family planning services?	41.	☐
42.	Do people in your area/locality, use modern methods of family planning (such as condoms, pills, IUDs, injectables, sterilization)?<br>[traditional methods-coitus interruptus, rhythm methods, abstinence, raw egg white viscous cervical mucus is fertile; after ovulation it is thick)	42.	☐
43.	If "YES", which methods are the most preferred methods in your locality? (3=spontaneous; 4=prompted)<br>Lactation Amenorrhoea Method<br>Progestin-Only Pill<br>Combined Oral Contraceptive Pill	43.	☐<br>☐<br>☐<br>☐

	Intrauterine Contraceptive Device<br>Injectable contraception (DMPA)<br>Implant<br>Male condom<br>Female condom<br>Natural family planning method<br>(coitus interruptus, rhythm methods, abstinence, cervical mucus)<br>Standard Days Method<br>Sterilization (Male)<br>Sterilization (Female)<br>Others, specify_______________		☐<br>☐<br>☐<br>☐<br>☐<br>☐<br>☐<br>☐<br>☐
**Subjective norms' influence on PPFP services**			
44.	Who influences your decisions about family planning practice?<br>Uncle<br>Friends<br>Parents<br>In-laws<br>Self<br>Husband<br>Doctors<br>Health worker	44.	☐<br>☐<br>☐<br>☐<br>☐<br>☐<br>☐<br>☐
45.	Other, please specify__________________	45.	___________________________________
46.	Does your husband support you in issues related to family planning services?<br>If "NO", skip to question 105	46.	☐
47.	How best does your husband assist you regarding	47.	

☐

	the use of family planning services?<br>Supporting through provision of transport<br>Reminding on dates of appointment<br>Gives items or finances<br>Use of condoms (both male and female condoms)		☐<br>☐<br>☐
48.	Any other, please specify___________________	48.	☐
49.	Whose opinion is important in your family concerning issues of reproduction?<br>Parents<br>In-laws<br>Self<br>Husband<br>Others	49.	☐<br>☐<br>☐<br>☐<br>☐
**Past experience and time of starting PPFP services**			
50.	Have you ever used any modern method of contraception within the past five (5) years such as condoms, pills, IUDs, injectables, sterilization?	50.	☐
51.	If No, why Not?<br>___________________________<br>___________________________<br>___________________________<br>___________________________<br>___________________________	51.	☐<br>☐<br>☐<br>☐<br>☐
52.	If YES, what method(s) of contraception did you use?<br>Lactation Amenorrhoea Method<br>Contraceptive pills/tablets	52.	☐<br>☐

☐
☐

	Progestin-Only Pill<br>Combined Oral Contraceptive Pill<br>Intrauterine Contraceptive Device<br>Injectable contraception (DMPA)<br>Implant<br>Male condom<br>Female condom<br>Natural family planning method<br>Standard Days Method<br>Sterilization (Male)<br>Sterilization (Female)<br>Others, specify________________		☐<br>☐<br>☐<br>☐<br>☐<br>☐<br>☐<br>☐<br>☐

An ancillary study to the WINGS on post partum contraceptive practices among urban and peri urban women in North India

53. Method	54. Did you experience any problem(s) with the method(s) you used	55. If "YES", what was the problem(s)? (11=painful; 12=uncomfortable, 13=increased bleeding, 14=husband did not like; 15=became weak, 16=gained weight, 17=painful coitus, 18=others, specify)	56. At what age of the last child did you start using the family planning method (mention in months)
Lactation Amenorrhoea Method	☐	☐☐	☐☐
Progestin-Only Pill	☐	☐☐	☐☐
Combined Oral Contraceptive Pill	☐	☐☐	☐☐
Intrauterine Contraceptive Device	☐	☐☐	☐☐
Injectable contraception (DMPA) Implant	☐	☐☐	☐☐
Male condom	☐	☐☐	☐☐
Female condom	☐	☐☐	☐☐
Natural family planning method	☐	☐☐	☐☐
Standard Days Method	☐	☐☐	☐☐
Sterilization (Male)	☐	☐☐	☐☐
Sterilization (Female)	☐	☐☐	☐☐
Others, specify	☐	☐☐	☐☐

57.	For how long did you have lactational amenorrhoea after you have given birth? Write in months; 77=did not have (Once woman gives the answer, this question will not be asked in the subsequent visit)	57.	☐☐
58.	Have you resumed sexual intercourse since the birth of your child?	58.	☐
59.	At what age of your child did you resume sexual intercourse? Write in months; 77=did not resume (Once woman gives the answer, this question will not be asked in the subsequent visit)	59.	☐☐
