# Supplementary material for: Postpartum contraceptive practices among urban and peri-urban women in North India: a mixed-methods cohort study protocol
Source: BMC Pregnancy Childbirth. 2021 Dec 10;21:820. doi: 10.1186/s12884-021-04294-3 (PMC8662907; doi:10.1186/s12884-021-04294-3)
Supplement: Supplementary file 2 — Additional file 2. [file 12884_2021_4294_MOESM2_ESM.pdf]

Qualitative Discussion Guides for Women, husbands and Health Care Providers

A. Qualitative In-depth Interview Topic Guide (Women)
Instruction: First go through the preliminaries and study information and consent form (Annex 1A)
Respondent's identification number: ……………………………………………………………….
Questionnaire administered by: ……………………………………………………………………..
Consent obtained? YES/NO……………………………………………………………………………..
Date: ………..

Topic 1. Current use of postpartum family planning (question numbers)

Which methods of contraception have you heard about?

Where did you hear about them?

How long ago did you give birth?

Are you currently using any method of contraception after you gave birth to your child?

If not, why not? (*Probe issues around unmet contraception need for postpartum family planning*)

If yes, which one? Why did you choose that one? (*Probe motivations for postpartum family planning*)

What do you like about the method you are using? What do you not like about it? (*Probe issues around postpartum family planning counselling and choice)*

How did you get the current method that you are using? What was the process? How easy or hard was it to get it? (*Probe issues around access to postpartum family planning)*

Was your partner/ husband involved in your decision regarding the use of postpartum family planning? If yes, in what way(s)? If not, why not?

Topic 2. Previous use of postpartum family planning and strategies for birth spacing

Have you ever used any other method of contraception soon after the birth of a child, if this is not your first child? (*Probe previous use experiences*)

If yes, which? What was your experience with using that/those method/methods after the birth of your previous child?

What other strategies have you used to space your children?

Topic 3. Previous experiences of unintended pregnancy

Have you ever conceived unintentionally? How did you manage the pregnancy?

What are your thoughts on abortion? What does your partner/husband have to say about it?

B. Qualitative In-depth Interview Topic Guide (Husbands)

Instruction: First go through the preliminaries and study information and consent form (Annex 1B)
Respondent's identification number: …………………………………………………………………
Questionnaire administered by: ……………………………………………………………………
Consent obtained? YES/NO………………………………………………………………………
Date: ………………………………………………………………………………………………

Topic 1. Ice breaking and rapport creation.

Tell me a bit about yourself.

How long ago did your wife give birth?

Topic 2: Knowledge and attitudes regarding post-partum family planning

What are the different methods women use to prevent pregnancy?

What are the different contraception methods used by men?

Is it ok for your wife to use family planning to avoid getting pregnant after giving birth? (*Probe*)

Is it ok for you to use family planning methods after your wife has given birth? (*Probe*)

Is it acceptable for a woman to avoid getting pregnant after they have had at least one child? (*Probe*)

Topic 3: Access to post-partum family planning

What could your wife do if she doesn't want to get pregnant? (*Probe*)

If your wife decided to avoid getting pregnant after birth of her child, how easy would it be for her to do so? (*Probe*) **How easy would it be for you to do so.**

How can your wife access the methods she needs to avoid pregnancy? (*Probe*)

How can you access the methods for family planning? (*Probe*)

What are the obstacles that she might face when she tries to avoid pregnancy? (*Probe*)

Topic 4: current practices on post-partum family planning

Are you currently using any method of contraception after you gave birth to your child?

If not, why not? (*Probe issues around unmet contraception need for postpartum family planning*)

If yes, what methods have you and your wife been using not to get pregnant?

Were you involved in the decision to use that methods, if yes how? Why did you choose that one? (*Probe men's involvement in decision making to use post-partum family planning)*

C. Qualitative In-depth Interview Topic Guide (Health Care Providers)

Instruction: First go through the preliminaries and study information and consent form (Annex 1C)
Respondent's identification number: ……………………………………………………………………
Questionnaire administered by: ………………………………………………………………………
Consent obtained? YES/NO……………………………………………………………………………
Date: ………

Topic 1: Knowledge and attitudes

When is it ok for a woman to get pregnant? (*Probe)*

Is it acceptable for a woman to avoid getting pregnant? (*Probe*

What could a woman do if she doesn't want to get pregnant?

What are the different methods women use to prevent pregnancy?

What are the different contraceptive methods men use?

Topic 2: Perceived access to post-partum family planning

If a woman decided to avoid getting pregnant, how easy would it be for her to do so?

How can a woman access the methods she needs to avoid pregnancy?

What are the obstacles that she might face when she tries to avoid pregnancy?

Topic 3: Perceived gaps and priorities to enhance post-partum family planning

Since you are providing your services in the area, what are the usual perceptions and practices in this community around contraception, particularly post-partum family planning practices (probe).

What can be done to increase women's access and use to **post-partum family planning**?
